# Enriched Environment Enhances Motor Function in Mice With Cerebral Infarction by Promoting mTOR‐Dependent Autophagy in the Contralateral Dentate Nucleus

**DOI:** 10.1002/kjm2.70167

**Published:** 2026-01-05

**Authors:** Chuan‐Jie Wang, Ke‐Wei Yu, Jun‐Fa Wu, Feng Tao

**Affiliations:** ^1^ Department of Rehabilitation Medicine, Jinshan Hospital Fudan University Shanghai China; ^2^ Department of Rehabilitation Medicine, Huashan Hospital Fudan University Shanghai China

**Keywords:** autophagy, cerebellar, enriched environment, motor function, stroke

## Abstract

Although environmental enrichment (EE) promotes post‐stroke motor recovery, the mechanisms underlying its regulation of cerebellar dentate nucleus (DN) plasticity remain unclear; this study therefore investigated how EE coordinates autophagy, mitochondrial homeostasis, and synaptic remodeling in the contralateral DN to facilitate functional restoration. Using a permanent middle cerebral artery occlusion (pMCAO) mouse model, we combined behavioral tests (rotarod and ladder rung) with electron microscopy, Western blotting (LC3B, p62, mTOR/p‐mTOR), and ELISA (TNF‐α, IL‐1β, IL‐6) to assess motor function, ultrastructural changes, autophagy, and neuroinflammation. Results demonstrated that EE significantly improved cerebellar‐mediated motor coordination, reduced neuronal degeneration, and preserved mitochondrial integrity, while enhancing autophagic activity via increased LC3B expression and decreased p62 accumulation. Ultrastructural analysis revealed elevated synaptic density and a shift toward mitochondrial rejuvenation, paralleled by attenuated neuroinflammatory responses and suppressed pro‐inflammatory cytokine levels, consistent with mTOR pathway inhibition. These findings indicate that EE promotes motor recovery by activating a self‐reinforcing repair loop in the DN, wherein autophagy‐mediated mitochondrial clearance and neuroinflammation suppression create a permissive microenvironment for synaptic remodeling, thereby establishing the DN as a pivotal hub for EE's therapeutic effects and supporting combinatory strategies targeting autophagy–mitochondrial pathways to optimize rehabilitation.

## 1 Introduction

The incidence of stroke has risen steadily over the years, leading to a significant prevalence of disability that severely impacts patients' daily lives. Motor impairment, affecting > 60% of survivors, remains the most debilitating sequela, severely compromising functional independence and quality of life [1]. Despite advancements in neurorehabilitation, ~50% of patients exhibit chronic motor deficits, underscoring the urgent need to elucidate novel recovery mechanisms.

Environmental enrichment (EE)—a paradigm leveraging multisensory stimulation to enhance experience‐dependent plasticity—has emerged as a promising strategy to promote post‐stroke recovery [2, 3]. In rodent models, EE typically involves enlarged housing equipped with running wheels, tunnels, and social cohorts, providing enhanced motor, cognitive, and social stimulation compared with standard conditions [4, 5, 6, 7, 8]. Research indicates that EE positively influences not only the peripheral areas affected by brain injury but also other brain regions, promoting mechanisms such as angiogenesis and synaptic remodeling that contribute to the recovery of damaged brain tissue and function. Nevertheless, the therapeutic efficacy and underlying mechanisms of EE require further investigation.

Previous research on stroke rehabilitation has primarily focused on the functional recovery of injured areas and local compensatory mechanisms, with less attention given to remote brain regions. Recent studies have underscored a strong association between the functionality of these remote brain regions and overall functional recovery following a stroke [9, 10]. For instance, studies utilizing proton nuclear magnetic resonance spectroscopy have indicated that a unilateral supratentorial stroke can inhibit the metabolism of the contralateral cerebellum—a phenomenon referred to as crossed cerebellar diaschisis (CCD). CCD is a common remote effect post‐stroke, characterized by reduced cerebral blood perfusion and metabolic activity in the contralateral cerebellum and, in severe cases, accompanied by cerebellar neuronal loss [11]. This suggests that a supratentorial stroke may cause remote damage to the contralateral cerebellum, raising questions about the influence of cerebellar function on recovery outcomes [12, 13]. Numerous studies have concluded that the cerebellum plays a critical role in the brain's functional reorganization after such strokes [14, 15]. Clinical trials have further demonstrated a significant positive correlation between motor function recovery in subacute stroke patients and the metabolic activity of the contralateral cerebellum [16]. Furthermore, deep brain stimulation of the cerebellar dentate nucleus (DN)—a deep‐seated nucleus within the cerebellum and the primary efferent pathway for cerebellar signal output, critically involved in regulating core functions, including motor coordination and motor learning—has shown promise in facilitating cortical functional reorganization and enhancing motor function [17]. With extensive fiber connections to the cerebral cortex, the DN serves as the primary nucleus for cerebellar signal output. If signal transmission between the cerebral cortex and the contralateral dentate nucleus is reduced or interrupted following a stroke, the structure and function of neuronal synapses in the DN may be affected, which could further impact motor function recovery. EE may ameliorate the CCD phenomenon, facilitate motor function restoration, and improve signal transmission between the cerebral cortex and cerebellar DN through a variety of external stimuli. The cerebellothalamocortical pathway is emerging as a promising target for neuromodulation aimed at enhancing rehabilitation outcomes. Optogenetic activation of the lateral cerebellar nucleus has also demonstrated the potential for long‐lasting functional recovery following a stroke [18], indicating that the cerebellum may serve as a vital target for neuromodulation strategies promoting functional recovery in mice following a supratentorial stroke.

Our previous studies have demonstrated that modulation of the cerebellar DN critically influences environmental enrichment (EE)‐induced motor recovery in mice with stroke, although the precise mechanisms remain unclear [19]. To address this knowledge gap, the current study employed EE intervention in a permanent middle cerebral artery occlusion (pMCAO) mouse model to systematically evaluate the therapeutic effects through behavioral assessments. We combined multilevel analyses, including Nissl staining for neuronal quantification in the contralateral cerebellar DN, ultrastructural examination of mitochondrial dynamics via electron microscopy, and molecular characterization using Western blotting and immunofluorescence to detect autophagy‐related proteins. This integrated approach enabled a comprehensive investigation of EE‐mediated recovery mechanisms at the morphological, subcellular, and molecular levels, with a particular focus on DN neuronal plasticity. Our findings aimed to elucidate the neurobiological basis of EE‐induced functional recovery in post‐stroke rehabilitation while providing novel therapeutic targets for clinical translation.

## 2 Materials and Methods

### 2.1 Animals

Male C57BL/6 mice (8–12 weeks old, weighing 22–25 g) were used in the experiments. All mice were housed in a controlled environment at 20°C with 45%–50% humidity and had *ad libitum* access to food and water. The lighting cycle lasted from 08:00 to 20:00. The Animal Ethics Committee at Fudan University approved all experimental methods used in the study (Shanghai, China). All possible measures were taken to minimize animal suffering.

### 2.2 Model of Permanent Middle Cerebral Artery Occlusion (pMCAO)

Isoflurane anesthesia was induced at 5% prior to pMCAO surgery and maintained at 1.5%–2% during the procedure. The left middle cerebral artery was occluded to induce focal cerebral ischemia. A midline incision was made on the ventral surface of the neck, followed by ligation of the left common carotid artery using a 6–0 silk suture. A 6–0 silk suture was also used to occlude the internal and external carotid arteries. A 4–0 surgical nylon monofilament with a silicone tip was inserted into the internal carotid artery through an opening in the proximal common carotid artery. The filament was positioned ~6.5 cm distal to the common carotid artery incision. The temperature was maintained at 36.5°C–37.5°C during the procedure using a controlled heating system. After surgery, mice were placed on a heating pad to recover from anesthesia before being returned to their cages. Sham‐operated mice underwent the same surgical procedure, except for the actual occlusion of the middle cerebral artery.

### 2.3 Animal Grouping and Housing Conditions

Surviving mice were assessed using beam balance tests 7 days after surgery [20]. Mice were placed on a beam (60 cm × 1.5 cm) for 1 min and scored between 0 and 6 as follows: steady balancing on the beam (score = 0); holding the side of the beam (score = 1); holding the beam with one limb slipped (score = 2); holding the beam with two limbs slipped (score = 3); falling off the beam within 40–60 s (score = 4); falling off the beam within 20–40 s (score = 5); and unable to stay on the beam (score = 6). Only mice with scores of 2–4 were considered successful models and included in this study. The successful models were then randomly divided into two groups: pMCAO mice with standard housing (SH; *n* = 10) and pMCAO mice with environmental enrichment (EE; *n* = 10). A third group consisted of sham‐operated mice housed under standard housing conditions (Sham, *n* = 10). After stroke induction, mice in the SH and sham groups were housed in conventional cages (294 mm × 190 mm × 130 mm) for 7–21 days. Mice in the EE group were housed in larger cages (545 mm × 395 mm × 200 mm) containing various enrichment items (such as chains, runners, ladders, pipes, and crates) that were exchanged every 3 days to maintain a stimulating and novel environment.

### 2.4 Behavioral Tests

All behavioral tests were conducted between 9:00 and 11:00 a.m. One hour before each test, the mice were acclimated to the laboratory environment. After completing all behavioral tests, the mice were returned to their respective environments for 60 min, after which they were euthanized, and brain tissue was extracted under aseptic conditions using 75% ethanol.

Rotarod Test: Motor performance was assessed using the rotarod method. To establish a performance baseline, a pre‐training test was conducted with linear acceleration (speed ranging from 5 to 40 rpm, steadily increasing over a 90‐s test period) for three consecutive days. The rotarod test was then performed on days 7, 14, and 21 after surgery. A maximum of 5 min was allowed between trials, with a 3‐min break provided to prevent animal exhaustion. The time taken by each mouse to fall off the rod during each trial was meticulously recorded, and the average latency was subsequently calculated as the outcome. The experiment was terminated when the mice completed three full rotations around the pole.

Ladder Rung Walking Test: The ladder‐rung device consisted of two plexiglass walls measuring 69.5 × 15 cm, positioned 5 cm apart to allow a mouse to pass through while preventing it from turning around. The holes in the walls were filled with 31 plastic bars, each with a diameter of 0.1 cm, spaced 1 cm apart. The entire device was elevated 17 cm above the ground and supported by two brackets. The mice were trained to ascend the ladder three times in succession prior to undergoing pMCAO surgery. The test was conducted on days 7, 14, and 21 after the surgical procedure. The limbs of walking mice were recorded simultaneously by positioning the camera at a slight ventral angle to the apparatus. Consequently, the overall steps and errors of the right forelimb were documented.

### 2.5 Nissl Staining Analysis

The instructions provided with the Nissl staining kit were followed precisely. The tissue sections were washed with Nissl differentiation solution and stained with cresyl violet solution for 45 min at room temperature (23°C ± 2°C). Tissue specimens were subsequently immersed in distilled water to halt the reaction. Cell morphological changes in the DN on the contralateral side of the infarction were then photographed at ×10 and ×20 magnifications. For each mouse, three non‐continuous slides were selected for quantitative analysis. The ImageJ plugin “Cell Counter” was utilized to quantify the number of living neurons in a 200 × 100 μm area of the DN.

### 2.6 Ultrastructural Analysis of Mitochondria and Synapses in Cerebellar DN

Four weeks post‐pMCAO, mice were deeply anesthetized with chloral hydrate (500 mg/kg, i.p.) and transcardially perfused with 0.9% saline followed by 4% paraformaldehyde. The contralateral cerebellar DN was dissected into 1 mm^3^ tissue blocks and post‐fixed in 2.5% glutaraldehyde (4°C, 24 h). Samples were dehydrated in graded ethanol, stained with 1% uranyl acetate, and embedded in Araldite resin. Ultrathin sections (65 nm) were cut using a diamond knife (Diatome), mounted on copper grids, and counterstained with lead citrate.

Mitochondrial morphology and synaptic density were analyzed using a Philips CM120 electron microscope at ×6000 magnification. For mitochondrial quantification, 15 non‐overlapping micrographs per animal were acquired, and intact mitochondria (defined by cristae visibility and double‐membrane integrity) were counted per 100 μm^2^ area.

$$\text{Synaptic density was calculated using the formula}:\mathrm{NV}=\frac{\mathrm{NA}}{d}$$

where *NV* = synapses per unit volume, *NA* = synaptic junctions per unit area, and *d* = mean synaptic junction length. Synapses were identified by the presence of presynaptic vesicles (> 2 vesicles) and postsynaptic densities. Junction lengths were measured using a calibrated graticule (0.1 mm increments) under 10× magnification. All analyses were performed blinded to the experimental groups.

### 2.7 Immunofluorescence Staining

Cerebellum slices were fixed in 4% paraformaldehyde for 10 min, followed by blocking with 10% BSA for 60 min at room temperature. After overnight incubation with primary antibodies (1:200), the specimens were washed three times with PBS and then incubated with fluorescence‐conjugated secondary antibodies for 60 min at room temperature. Fluorescence images were captured using an inverted fluorescence microscope (Leica, Wetzlar, Germany), and the integrated optical density was calculated using Image‐Pro Plus 6.0 (MediaCybernetics Inc., MD, USA).

### 2.8 Western Blotting

The contralateral cerebellar DN was dissected 21 days post‐pMCAO and homogenized in RIPA lysis buffer (containing protease/phosphatase inhibitors). Protein concentrations were determined via BCA assay (Pierce), and samples were denatured at 95°C for 10 min in Laemmli buffer. Equal amounts of protein (20 μg) were separated on 10% SDS‐PAGE gels (Bio‐Rad) and transferred onto nitrocellulose membranes (0.2 μm pore size, Bio‐Rad). Membranes were blocked with 5% nonfat milk in PBST (0.1% Tween‐20) for 1 h at RT, followed by overnight incubation with primary antibodies at 4°C. Primary Antibodies included anti‐LC3B (rabbit, 1:1000, L754, Sigma), anti‐p62 (rabbit, 1:1000, 18420‐1‐AP, PTG), anti‐mTOR (rabbit, 1:1000, #2983, CST), anti‐p‐mTOR (rabbit, 1:1000, #5536, CST), mouse monoclonal anti‐GAP‐43 (1:2000, #WH0002596M1, Sigma), mouse monoclonal anti‐synaptophysin (Mouse, 1:1000, #SAB4200476, Sigma), mouse monoclonal anti‐PSD‐95 (1:1000), and mouse monoclonal anti‐β‐tubulin (1:1000, #SAB4200081, Sigma). After three 15‐min PBST washes, the membranes were incubated with HRP‐conjugated secondary antibodies (goat anti‐rabbit, 1:5000, #A6154, Sigma; Goat anti‐mouse, 1:5000, #A4416, Sigma) for 1 h at room temperature. Protein bands were visualized using ECL Prime (Amersham) and quantified densitometrically using ImageJ software (NIH). Target protein levels were normalized to GAPDH and β‐tubulin.

### 2.9 Enzyme‐Linked Immunosorbent Assay (ELISA)

DN tissues from the cerebellum were mechanically homogenized in 0.9% normal saline at a concentration of 200 mg/mL and centrifuged at 12,000 rpm for 10 min at 4°C. Following the manufacturer's instructions, specialized ELISA kits for mice (Elabscience Biotechnology, China) were employed to measure the levels of IL‐1β, IL‐6, and TNF‐α in brain tissue homogenates. Final cytokine concentrations were calculated using optical density measurements.

### 2.10 Statistical Analysis

Calculations were performed using SPSS version 25.0. All data are presented as mean ± standard error of the mean (SEM). Data from motor behavioral tests were analyzed using two‐way ANOVA, followed by Tukey's multiple comparisons post hoc test. One‐way ANOVA and Fisher's least significant difference post hoc tests were used to evaluate the western blot analysis, ELISA, Nissl staining, and mitochondrial count results. Correlations between motor function outcomes and p‐mTOR, LC3B, and p62 levels were analyzed using Prism version 8.0.1 (GraphPad Software, La Jolla, CA, USA) with Spearman's correlation coefficients. Statistical significance was determined at *p* ≤ 0.05.

## 3 Results

### 3.1 EE Promotes the Recovery of Motor Functions After pMCAO

**FIGURE 1:**
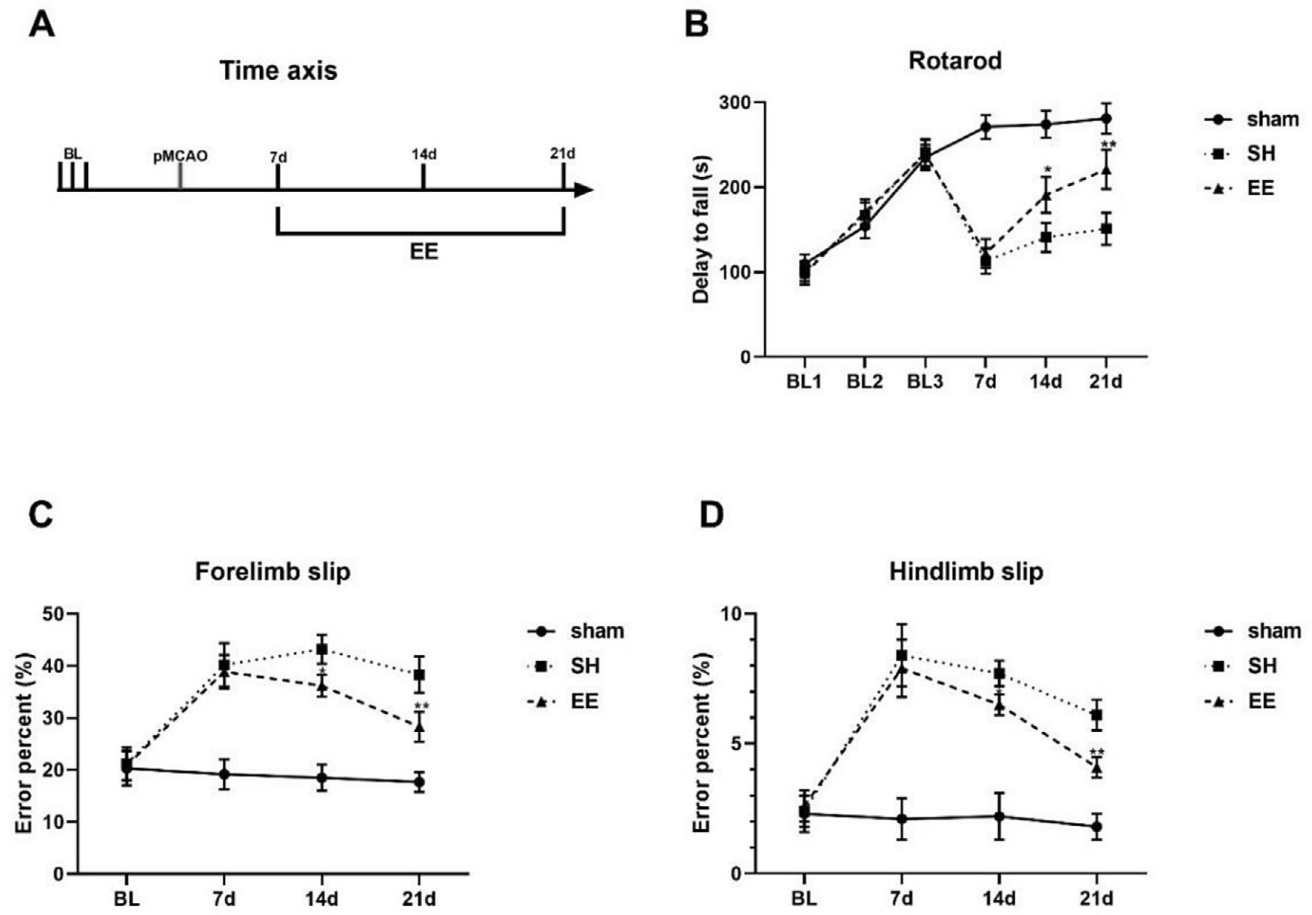
Effects of EE on motor recovery after pMCAO. (A) Timeline of behavioral tests. Motor assessments included the rotarod and ladder rung walking tests. BL: Baseline data for the rotarod test. (B) Time course of latency to fall from the rotarod. (C, D) Percentage of limb slips in the ladder rung walking test for both forelimbs and hindlimbs. Data are presented as mean ± standard error of the mean (SEM). *SH versus EE group; *p ≤ 0.05; **p ≤ 0.01.

At baseline, there were no significant differences in motor abilities among the three groups. On day 7 post‐pMCAO‐induced stroke, severe motor deficits were observed in the SH and EE groups compared to the sham group (Figure 1). The EE group outperformed the SH group in the rotarod test on both day 14 (*p* < 0.05) and day 21 (*p* < 0.01) post‐pMCAO, as EE commenced on day 7 after stroke. The EE group also demonstrated significantly better performance in the ladder rung walking test than the SH group on both day 14 (*p* < 0.05) and day 21 (*p* < 0.01) post‐pMCAO. The number of forelimb slips (Figure 1C) and hindlimb slips (Figure 1D) was significantly lower in the EE group than in the SH group. Overall, compared with skilled motor functions mediated by the cortex (ladder rung walking test), the EE intervention was more effective in enhancing balance and coordination functions mediated by the cerebellum (rotarod test).

### 3.2 EE Reduces the Neurons Lost in the Contralateral DN

**FIGURE 2:**
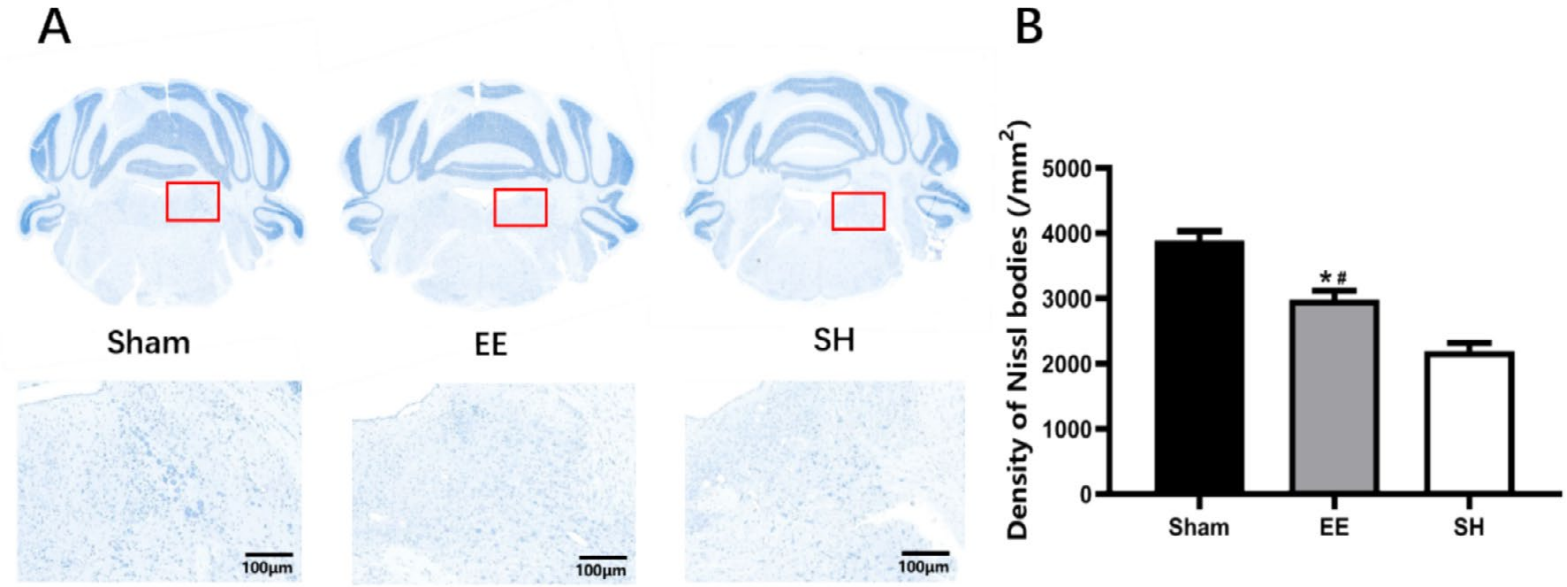
EE reduces the neurons lost in the contralateral cerebellar DN. (A) Nissl staining of the mouse cerebellar DN in the Sham, EE, and SH groups. Red rectangles indicate the dentate nucleus (DN); images below show ×400 magnification of Nissl‐stained neurons in the DN. (B) Quantitative analysis of neuronal density in representative areas of interest. Data are presented as mean ± standard error of the mean (SEM), with *n* = 10 per group. *p < 0.05, EE and SH groups versus Sham group. #*p* < 0.05, EE group versus SH group.

Compared with the Sham group, the number of Nissl‐stained neurons in the contralateral DN of mice with cerebral infarction was significantly lower in both the EE and SH groups (*p* < 0.05). However, the EE group exhibited a significant increase in Nissl bodies in the contralateral DN after infarction compared with the SH group (*p* < 0.05) (Figure 2).

### 3.3 EE Reduced Mitochondrial Loss in the Cerebellar DN

**FIGURE 3:**
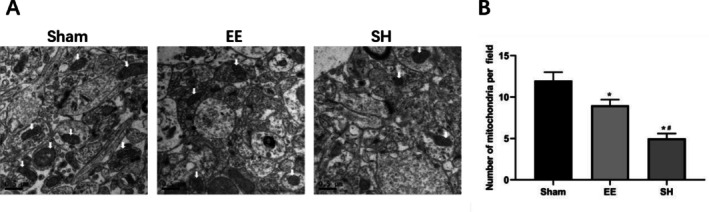
EE reduces mitochondrial loss in the contralateral cerebellar DN. (A) Electron micrographs (×6000 magnification) showing mitochondria in the contralateral DN of the Sham, EE, and SH groups. (B) Quantification of mitochondria in the contralateral DN among the three groups. Data are presented as mean ± standard error of the mean (SEM), with *n* = 10 per group. *p < 0.05, EE and SH groups versus Sham group. #*p* < 0.05, SH group versus EE group.

Electron microscopy analysis revealed that neurons in the contralateral cerebellar DN of the EE and SH groups contained significantly fewer mitochondria than those in the sham group. Conversely, the contralateral cerebellar DN neurons in the EE group exhibited a significantly higher number of mitochondria than those in the SH group (Figure 3).

### 3.4 EE Enhances Autophagy of Contralateral DN Neurons

**FIGURE 4:**
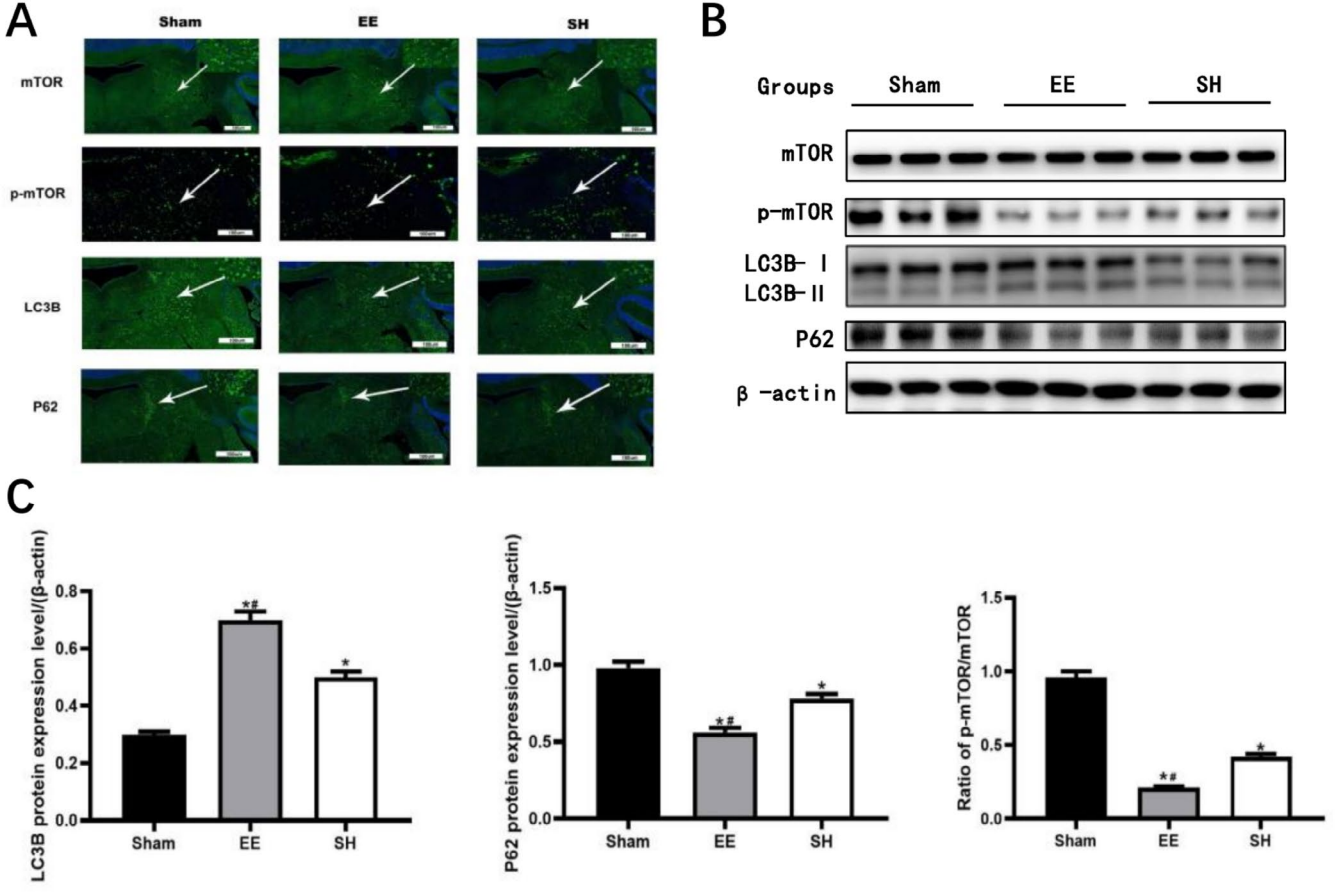
EE enhances autophagy of contralateral DN neurons. (A) Representative immunofluorescence images of the contralateral DN in the EE, SH, and Sham groups. Scale bar = 100 μm. (B) Representative western blot images of the contralateral DN in the EE, SH, and Sham groups. (C) The bar graph depicts quantification of protein expression of the contralateral DN across different groups. Data are presented as mean ± standard error of the mean (SEM), with *n* = 10 per group. *p < 0.05, EE and SH groups versus Sham group. #*p* < 0.05, EE group versus SH group.

The expression of p‐mTOR, LC3B, and p62 in the contralateral DN was quantitatively analyzed using immunofluorescence labeling and western blotting to assess autophagy levels. Compared with the Sham group, both the SH and EE groups exhibited a marked decline in the p‐mTOR/mTOR ratio, a substantial reduction in p62 levels, and a significant increase in LC3B, as determined by immunofluorescence imaging and western blot analyses. Compared with the SH group, the EE group demonstrated a considerable decline in the p‐mTOR/mTOR ratio, a significant decrease in p62 levels, and a significantly higher level of LC3B. The expression level of β‐actin served as a loading control for the aforementioned analyses (Figure 4).

### 3.5 Correlation of Motor Function Outcomes With p‐mTOR, LC3B, and P62

**FIGURE 5:**
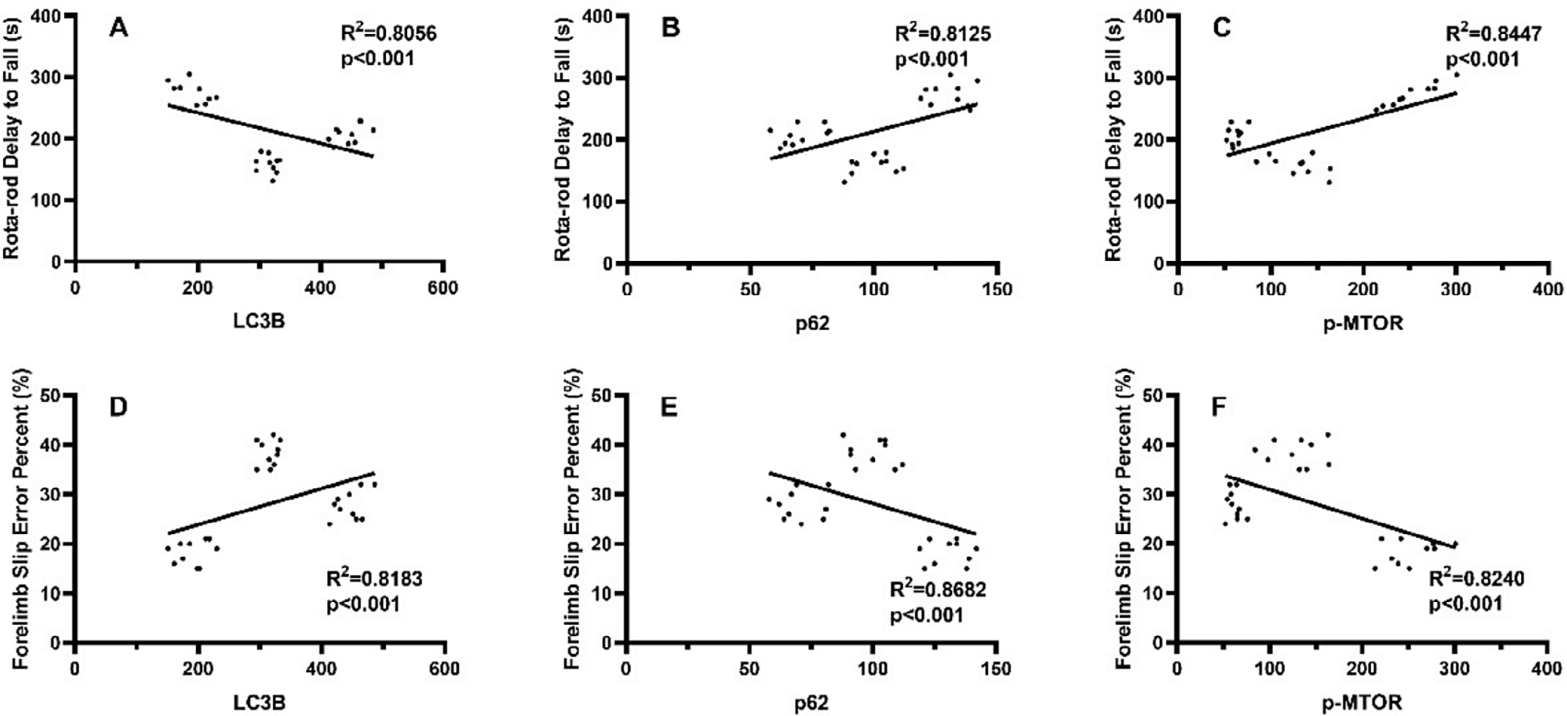
Correlation of motor function with the relative expression of LC3B, p62, and p‐mTOR. (A–C) Correlation of rotarod delay to fall time with LC3B, p62, and p‐mTOR; (D–F) Correlation of forelimb slip error percentage with LC3B, p62, and p‐mTOR; *R* ^2^ represents Spearman's correlation coefficient. *R* ^2^ > 0.7 was considered a significant correlation.

To assess the correlation between motor function outcomes (measured on post‐modeling day 21) and the levels of p‐mTOR, LC3B, and p62 in post‐stroke mice, Spearman's correlation coefficients (*R* ^2^) were calculated. The analysis revealed that the duration of rotarod delay was positively correlated with the relative expression of LC3B (*R* ^2^ = 0.7538, *p* < 0.001), but negatively correlated with the relative expression of p62 (*R* ^2^ = 0.7961, *p* < 0.001) and p‐mTOR (*R* ^2^ = 0.7571, *p* < 0.001). Additionally, the forelimb slip error percentage was inversely correlated with LC3B expression (*R* ^2^ = 0.8091, *p* < 0.001), but positively correlated with p62 (*R* ^2^ = 0.7714, *p* < 0.001) and p‐mTOR (*R* ^2^ = 0.7731, *p* < 0.001) (Figure 5).

### 3.6 EE Increase Synaptic Density of the Contralateral Cerebellar DN

**FIGURE 6:**
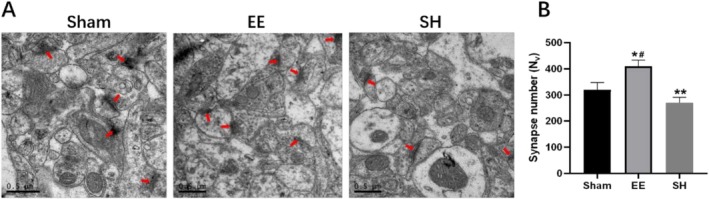
Synaptic density in the contralateral cerebellar dentate nucleus region of mice following pMCAO. (A) Electron microscopy image of the contralateral cerebellar dentate nucleus area after infarction; red arrows indicate synapses. (B) Statistical analysis of the number of synapses in each group: **p < 0.05, SH versus Sham; *p < 0.001, EE versus Sham; #*p* < 0.001, EE versus SH.

Synaptic changes were assessed using transmission electron microscopy. Electron microscopy images showed significant differences in the number of synapses in the cerebellar dentate nucleus area opposite the infarction among the three groups (SH = 287.37 ± 17.40, EE = 395.13 ± 34.73, Sham = 327.63 ± 36.76, *p* < 0.000, F (2, 21) = 24.878, *n* = 8). Further pairwise comparisons revealed a decrease in the number of synapses in the SH group compared to the Sham group (*p* = 0.016) and a significant increase in the number of synapses in the EE group compared with both the SH and Sham groups (*p* < 0.001) (Figure 6).

### 3.7 Environmental Enrichment Reduces Inflammatory Reactions in the Contralateral DN

**FIGURE 7:**
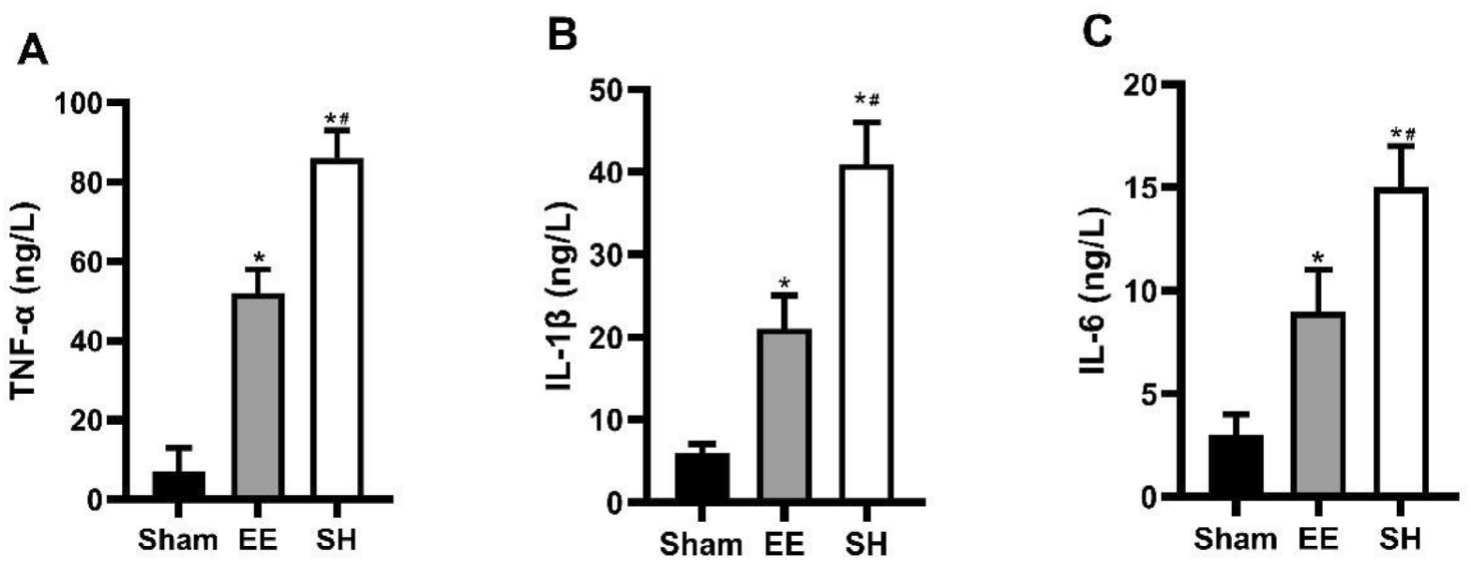
Environmental enrichment reduces the inflammatory reaction in the contralateral cerebellar dentate nucleus (DN). Comparison of TNF‐α (A), IL‐1β (B), and IL‐6 (C) expression among the three groups in the contralateral DN. Data are presented as the mean ± standard error of the mean (SEM), with *n* = 10 per group. *p < 0.05 indicates a significant difference between the EE and SH groups compared to the Sham group. #*p* < 0.05 indicates a significant difference between the SH group and the EE group.

The degree of inflammation was assessed by quantifying the expression levels of TNF‐α, IL‐1β, and IL‐6 in the contralateral cerebellar DN using ELISA. The SH and EE groups exhibited significantly higher expression levels of TNF‐α, IL‐1β, and IL‐6 compared with the Sham group. However, the expression levels of TNF‐α, IL‐1β, and IL‐6 in the EE group were significantly lower than those in the SH group (Figure 7).

## 4 Discussion

Our findings provide preliminary evidence that environmental enrichment (EE) improves poststroke motor function through multifaceted interactions in the contralateral cerebellar dentate nucleus (DN), with autophagy activation emerging as a central mechanism. EE selectively enhanced cerebellar‐mediated coordination, as evidenced by improved performance in balance‐related tasks, while concurrently mitigating DN neurodegeneration through mitochondrial preservation and restoration of synaptic density. The observed suppression of pro‐inflammatory cytokines (TNF‐α, IL‐1β, IL‐6) in the DN suggests a potential anti‐inflammatory role of EE, though this requires further validation. Although these effects—enhanced autophagic flux, structural plasticity, and inflammation modulation—appear to be temporally correlated, their causal relationships remain speculative. Notably, the parallel improvements in mitochondrial integrity and synaptic connectivity may reflect autophagy's dual role in cellular detoxification and microenvironmental regulation, providing a plausible framework for the therapeutic effects of EE. These exploratory results position the DN as a key site for EE‐driven neurorepair, warranting deeper investigation into how autophagic pathways interface with inflammatory and synaptic remodeling processes during post‐stroke recovery.

There are extensive fiber connections between the cerebellum and the brain. In addition to its association with the motor cortex, the deep cerebellar nuclei also maintain close bidirectional communication with other brain regions, such as the premotor cortex and parietal cortex [21]. Recently, the relationship between cerebellar function and motor recovery after stroke has garnered significant attention. An animal study found that low‐intensity focused ultrasound stimulation of the deep cerebellar regions aids in the recovery of hemispheric balance in mice after ischemic stroke [22]. Wang et al. found that fastigial nucleus stimulation alleviates post‐stroke depression in a rat model of chronic unpredictable mild stress [23]. These findings suggest that cerebellar function may influence the functional reorganization of the brain following a stroke. Increasing evidence suggests that the loss of function in local brain regions after stroke cannot fully account for the functional impairments observed in patients. Disruptions in neural network connections and subsequent remote neurodegeneration may be important mechanisms underlying multiple symptoms in these patients [24]. Stroke, especially in the middle cerebral artery region, often leads to crossed cerebellar diaschisis (CCD), which is characterized by reduced cerebral blood flow and energy metabolism in the contralateral cerebellar hemisphere. Our research revealed that unilateral middle cerebral artery occlusion causes a decrease in Nissl bodies and loss of mitochondria in the contralateral DN, and that EE intervention can significantly ameliorate this condition. Moreover, EE intervention significantly increased the scores in the rotorod and ladder rung walking tests in the modeled mice, indicating that EE preferentially enhances cerebellar‐dependent motor coordination.

Autophagy plays a crucial role in clearing damaged cellular components and maintaining neuronal stability, particularly in response to ischemic stress [25]. Under post‐stroke conditions, the upregulation of autophagy‐related proteins is observed as a protective response to minimize damage and maintain cellular integrity [26, 27]. Our study found that EE intervention increased autophagy‐related protein levels in the contralateral DN, which correlated positively with improved motor function. This finding aligns with recent studies suggesting that autophagy contributes to neuroplasticity by facilitating the removal of cellular debris and supporting synaptic remodeling in regions indirectly affected by the stroke [2, 28].

Mitochondria serve as the central hubs of cellular energy production and are acutely vulnerable to ischemic damage [29, 30], with stroke‐induced dysfunction amplifying neuronal energy deficits and oxidative stress, thereby exacerbating post‐injury neurodegeneration [31]. Our findings demonstrate that environmental enrichment (EE) counteracts this degenerative cascade by activating a selective autophagic response in the dentate nucleus (DN). Elevated LC3B‐II levels, coupled with reduced p62 expression, indicate enhanced autophagic flux, which preferentially targets damaged mitochondria for clearance while preserving functional populations. This selective mitochondrial quality control aligns with the established role of autophagy in maintaining neuronal homeostasis, in which balanced organelle turnover supports cellular energy stability [32, 33, 34]. Notably, the morphological integrity of mitochondria—characterized by intact cristae structure and absence of swelling—directly correlates with synaptic density. Transmission electron microscopy studies have shown that age‐related mitochondrial damage (e.g., cristae fragmentation) coincides with reduced synaptic number and degenerative synaptic architecture [35]. Mechanistically, EE's effects may be mediated through modulation of the mTOR pathway, a key regulator of autophagy initiation and mitochondrial biogenesis [36, 37].

Concurrently, EE promotes synaptic preservation and reorganization in the DN, as evidenced by ultrastructural analysis revealing increased synaptic density. The coexistence of mitochondrial integrity and synaptic plasticity suggests a potential interdependency, wherein autophagy‐mediated energy recovery creates a metabolically supportive microenvironment for neural circuit remodeling. Mechanistically, EE may maintain synaptic structural integrity by promoting mitochondrial regeneration, particularly through LC3B‐upregulated mitophagy, which clears damaged organelles while preserving functional mitochondria—an observation consistent with studies showing that spatially stable mitochondrial compartments fuel the local protein synthesis required for synaptic plasticity [38]. This aligns with previous research demonstrating that improved mitochondrial function enhances synaptic plasticity via ATP‐dependent neurotransmitter release and the regulation of calcium homeostasis [39]. Although the precise molecular crosstalk between autophagic activity and synaptic reorganization requires further investigation, this dual effect underscores EE's capacity to coordinately address multiple facets of post‐stroke pathology. Such integrated repair mechanisms are consistent with observations in other energy‐sensitive neural systems, where autophagy sustains structural plasticity by harmonizing organelle homeostasis with synaptic maintenance.

Furthermore, EE's anti‐inflammatory effects in the DN, marked by TNF‐α, IL‐1β, and IL‐6 reduction, add a novel layer to its neuroprotective profile. While the mTOR pathway is implicated in both autophagy and inflammation [28, 40], our results suggest that EE may suppress neuroinflammation through mTOR‐dependent and/or‐independent mechanisms—a hypothesis that requires validation via cell‐type‐specific analyses (e.g., microglial mTOR knockout models).

Our findings contribute to an emerging understanding of autophagy and mitochondrial function as interconnected therapeutic targets for post‐stroke recovery. Modulation of these pathways may provide new avenues for enhancing neuroprotection and neuroregeneration after stroke. This study supports future investigations into how EE and other therapeutic strategies could synergistically target autophagy and mitochondrial pathways to facilitate motor recovery. Developing combined protocols that leverage EE with pharmacological modulators of the autophagy‐mitochondrial axis could maximize stroke recovery outcomes.

## 5 Scientific and Clinical Significance

This study highlights the critical roles of autophagy and mitochondrial function in post‐stroke recovery, suggesting that EE‐based interventions may offer an accessible approach to support cellular repair mechanisms. By reinforcing mitochondrial stability and enhancing autophagic processes, EE not only promotes neural survival but also aids in restoring motor functions, making it a promising adjunct therapy in stroke rehabilitation. Further research is warranted to elucidate the specific pathways involved and to explore the potential of combining EE with pharmacological agents to optimize neurofunctional outcomes.

## 6 Strengths and Limitations of the Study

This study had two primary strengths. First, it distinguishes itself from studies focusing on the stroke area and its surrounding regions by exploring the contralateral DN, which connects to the affected area through multiple synaptic pathways, thereby providing new insights for stroke treatment. Second, it used EE as a therapeutic intervention to investigate its impact on remote areas, which aided in clarifying the mechanisms through which EE operates.

This study also has limitations. First, the physical activity of EE mice (e.g., via accelerometers) and sleep–wake cycles were unmonitored; therefore, the link between EE‐induced activity increases and motor recovery relies on indirect literature evidence. While prior studies show that EE does not disrupt rodent sleep, sleep‐EE therapeutic interactions cannot be fully excluded. Second, the absence of autophagy blockers (e.g., pharmacological inhibitors) limits autophagy conclusions. In addition, other EE‐related mechanisms affecting contralateral DN function after supratentorial stroke should be explored. Future studies should address these issues to strengthen causal inferences regarding EE in post‐stroke recovery.

## 7 Conclusions

In conclusion, this study demonstrates that EE may alleviate remote damage in contralateral DN neurons following supratentorial stroke by enhancing mTOR‐dependent autophagy. This study suggests that preventing remote damage should be a key focus in rehabilitation strategies following brain injury.

## Funding

Our study was supported by the National Natural Science Foundation of China, Nos. 81972140 (to J.F.W.), the National Natural Science Foundation of China, Nos. 82072547 (to K.W.Y.), and the Youth Research Start‐up Fund of Jinshan Hospital, Nos. JYQN‐JC‐202205 (to F.T.).

## Ethics Statement

The Institutional Animal Care and Use Committee of Fudan University approved the experimental protocols. The ethical agreement number is 2022 Jinshan Hospital JS‐025.

## Conflicts of Interest

The authors declare no conflicts of interest.

## Data Availability

The data that support the findings of this study are available on request from the corresponding author. The data are not publicly available due to privacy or ethical restrictions.
